# S100A9, as a potential predictor of prognosis and immunotherapy response for GBM, promotes the malignant progression of GBM cells and migration of M2 macrophages

**DOI:** 10.18632/aging.205949

**Published:** 2024-08-13

**Authors:** Qiankun Ji, Zibo Li, Yazhou Guo, Xiaoyang Zhang

**Affiliations:** 1 Department of Neurosurgery, Zhoukou Central Hospital, Zhoukou, Henan 466000, P.R. China

**Keywords:** S100A9, bioinformatics, glioblastoma (GBM), M2 macrophages, tumor microenvironment

## Abstract

In the past decades, the therapeutic effect of glioblastoma (GBM) has not been significantly improved. Generous evidence indicates that S100A9 has a wide range of functions in tumors, but its exploration in GBM is less. The purpose of this study is to conduct a comprehensive bioinformatics analysis and cytological experiment on S100A9 in GBM. The expression data and clinical data of GBM samples were downloaded from the public database, and comprehensive bioinformatics analysis was performed on S100A9 in GBM using R software. Wound healing assay and transwell assay were used to detect the migration activity of cells, and colony formation assay, EdU staining, and CCK-8 assay were used to detect the proliferation activity of cells. The effect of S100A9 on the migration activity of M2 macrophages was verified by the cell co-culture method. The protein expression was detected by western blotting and immunohistochemical staining. S100A9 is an independent prognostic factor in GBM patients and is related to poor prognosis. It can be used as an effective tool to predict the response of GBM patients to immune checkpoint inhibitors (ICIs). In addition, S100A9 can promote the malignant progression of GBM and the migration of M2 macrophages. On the whole, our study highlights the potential value of S100A9 in predicting prognosis and immunotherapeutic response in GBM patients. More importantly, S100A9 may promote the malignant progress of GBM by involving in some carcinogenic pathways and remodeling the tumor microenvironment (TME).

## INTRODUCTION

As the most lethal tumor in the central nervous system, the treatment of glioma still faces many obstacles, and all the previous treatments have failed to achieve satisfactory results [1]. Although immunotherapy as the new treatment method has achieved the obvious curative effect in various tumors [2, 3], the special immune microenvironment in glioma hindered the application of immunotherapy [4]. Therefore, strengthening the study of the glioma immune microenvironment is the key prerequisite to improving the application of immunotherapy in glioma. In addition, clarifying the mechanism of various factors affecting the immune microenvironment of glioma can not only provide an important theoretical basis for improving glioma immunotherapy strategies but also provide a new theoretical basis for revealing the internal causes of glioma malignant progress.

Numerous studies on the tumor microenvironment (TME) have confirmed the importance of non-tumor cells in maintaining tumor growth and response to Immune Checkpoint Inhibitors (ICIs) [5, 6]. As a component of TME, tumor-associated macrophages (TAMs) account for about 50% of the total non-cancer glioma cell population and actively participate in promoting tumor malignant progression [7]. In recent years, the blood-brain barrier (BBB) has been regarded as the main reason for the failure of immunotherapy and other treatment methods for glioma. It is worth emphasizing that there is evidence that macrophages have the natural ability to cross the BBB, and the malignant degree of glioma is related to the number of infiltrating myeloid cells, which are composed of microglia and macrophages [8, 9].

S100A9, as a member of the danger-related molecule family, is induced during infection, injury, or inflammation to initiate the initial rapid inflammatory response [10, 11]. It accounts for about 45% of the cytoplasmic protein in neutrophils and 5% in monocytes [12, 13]. S100A9 has a wide range of functions, which not only regulate the calcium homeostasis in myeloid cells but also can be secreted into the extracellular environment to affect like cytokines and deeply involved in the development of inflammation [12, 14–16]. In GBM, the expression of S100A9 in myelogenous suppressor cells was significantly increased [17], and this change was observed in a variety of tumors, which was believed to induce changes in tumor immune microenvironment leading to immunosuppression [14, 18]. Meanwhile, the expression of S100A9 was also found to increase in glioma stem cells and promoted their proliferation [19]. The amount of S100A9 in the blood of glioma patients was also increased, which can be regarded as a prognostic biomarker of glioma [20].

At present, the research of S100A9 in tumors mainly focuses on the impacts on immune cells and the microenvironment. S100A9 not only played an important role in myeloid cells but also worked as an exocrine protein in chemotaxis and aggregation of immune cells [10]. Meanwhile, we found that S100A9 was also expressed in GBM cells. At present, there is no detailed analysis of the specific role of S100A9 in GBM cells, which is the focus of our study.

In this study, we found that S100A9 did have significant differences in transcriptional expression between GBM tissues and normal brain tissues by analyzing the public GBM RNA-seq database (TCGA, CGGA, etc.,) and this difference was also reflected in the protein level. Through bioinformatics analysis, we found that the expression of S100A9 in GBM tissues had prognostic significance for patients. Furthermore, we determined the possible role of S100A9 in GBM through enrichment analysis. Cytological experiments *in vitro* showed that increasing the expression of S100A9 in GBM cells can promote cell proliferation and migration. In addition, we found that S100A9 was associated with most immune pathways and immune markers, and it is also an effective predictor of immunotherapeutic response to GBM. It was also verified by cell co-culture experiment that S100A9 could promote the migration of M2 macrophages, indicating that S100A9, as an exocrine protein of GBM cells, was helpful to the recruitment of M2 macrophages.

## RESULTS

### Differential analysis of S100A9 in pan-cancer

**Figure 1:**
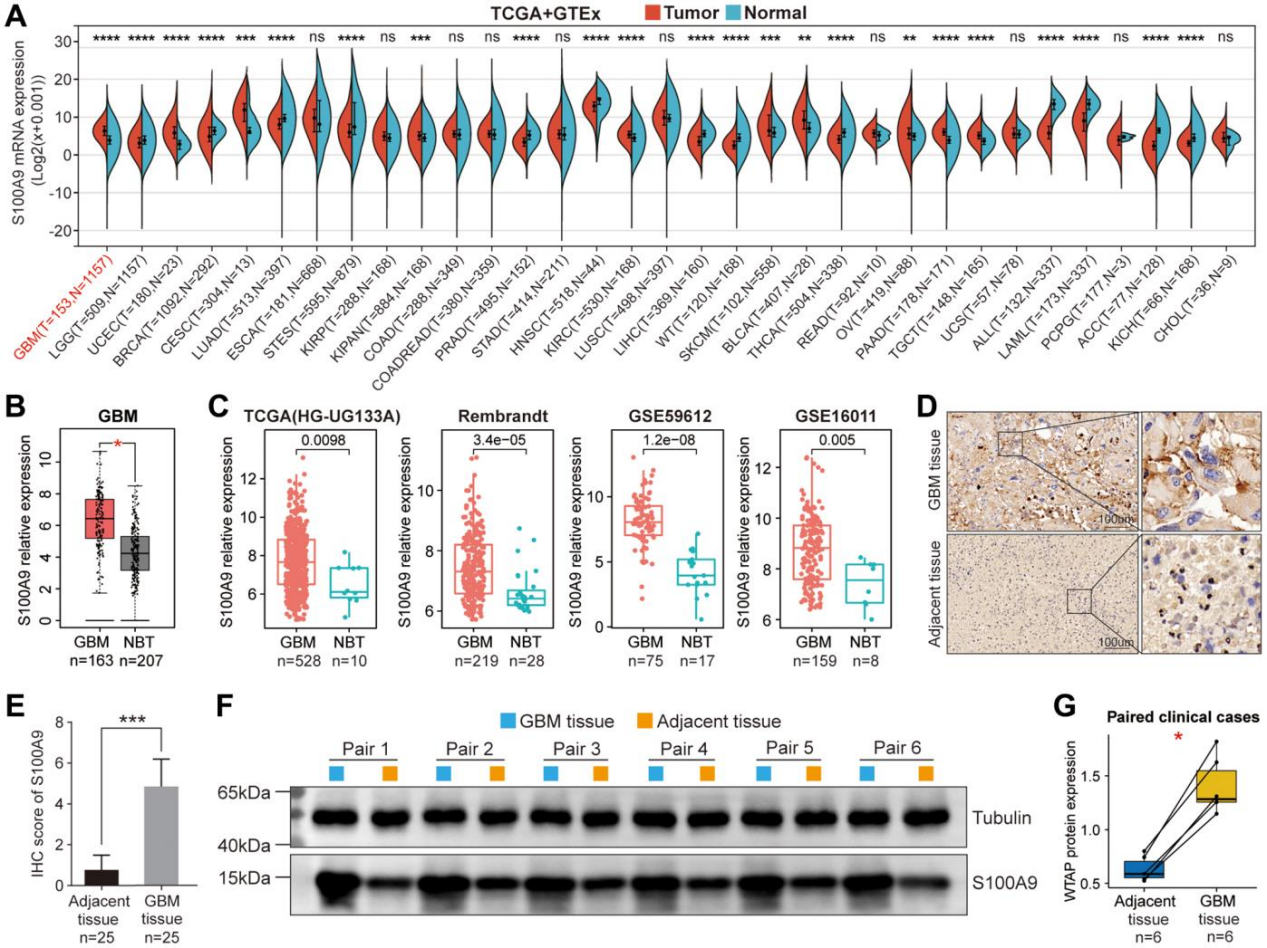
**Differential expression analysis of S100A9.** (**A**) Differential expression of S100A9 between tumor tissues and normal tissues in pan-cancer. (**B**) Differential expression of S100A9 between GBM tissues and NBTs in the TCGA database. (**C**) Differential expression analysis of S100A9 in four independent GBM cohorts. (**D**, **E**) Immunohistochemical staining of 25 pairs of GBM tissues and corresponding adjacent tissues. (**F**, **G**) Western blotting detection of six pairs of GBM tissues and corresponding adjacent tissues.

**Figure 2:**
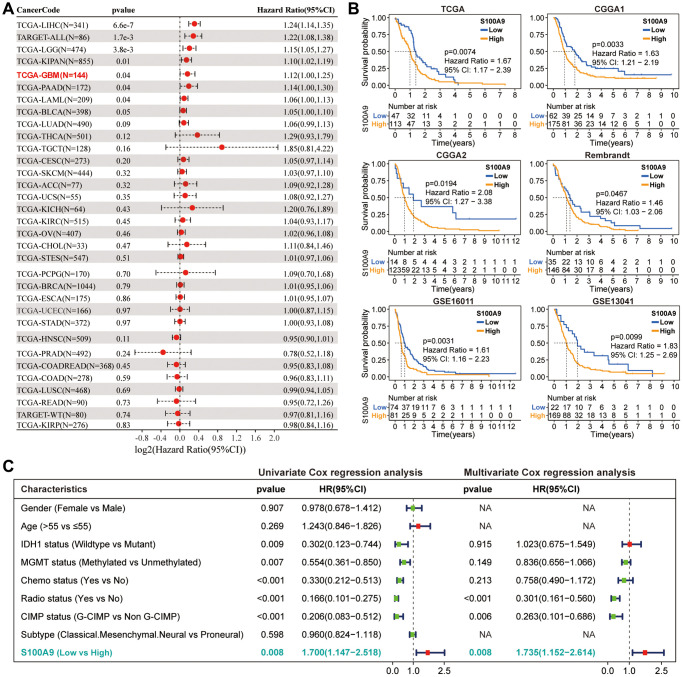
**Prognostic analysis of S100A9.** (**A**) Univariate Cox regression analysis was used to explore the prognostic value of S100A9 from the perspective of pan-cancer. (**B**) K-M survival curves of S100A9 in six independent GBM cohorts indicated that GBM patients with low S100A9 expression tend to have better survival outcomes. (**C**) Univariate and multivariate Cox regression analysis of different clinicopathological characteristics and S100A9 in TCGA cohort.

According to the analysis results of the Sangerbox3.0 platform, there is a significant difference in the expression level of S100A9 mRNA in pan-cancer, which is higher in GBM, UCEC, CESC, KIPAN, KIRC, SKCM, BLCA, OV, PAAD, and TCGT datasets, and lower in LGG, BRCA, LUAD, STES, PRAD, HNSC, LIHC, WT, THCA, ALL, LAML, ACC and KICH datasets (Figure 1A). Abbreviations of various cancers in pan-cancer are listed in Supplementary Table 1. In addition, whether S100A9 mRNA can be used as a risk factor to affect the survival of cancer patients in pan-cancer also varies, which can be used as a risk factor to affect the survival of cancer patients in LIHC, ALL, LGG, KIPAN, GBM, PAAD, LAML and BLCA datasets (Figure 2A).

### S100A9 is upregulated in GBM and indicates poor prognosis

Based on the pan-cancer analysis, we further explored the differential expression and prognosis of S100A9 in GBM. The analysis on the GEPIA website revealed that compared with normal brain tissues (NBTs), the expression level of S100A9 mRNA in GBM tissues was remarkably up-regulated (Figure 1B). In four independent GBM cohorts (TCGA (HG-UG133A), Rembrandt, GSE59612, and GSE16011), the upregulation of S100A9 mRNA was also verified, including 528, 219, 75, and 159 cases of human GBM tissues and 10, 28, 17, and 8 cases of NBTs, respectively (Figure 1C). Immunohistochemical staining analysis of 25 pairs of GBM tissues and their corresponding adjacent tissues illustrated that the S100A9 protein level in GBM tissues was significantly increased (Figure 1D, 1E). In addition, the upregulation of S100A9 protein in GBM tissues was also verified in six pairs of GBM tissues and their corresponding adjacent tissues (Figure 1F, 1G). Compared with patients with low S100A9 expression, patients with high S100A9 expression tended to have shorter survival (Figure 2B). Furthermore, through univariate and multivariate Cox regression analysis, we determined that S100A9 was an independent prognostic factor for GBM patients (Figure 2C). The clinicopathological characteristics of GBM patients in TCGA cohort and the relationship between clinicopathological characteristics and S100A9 expression are listed in the Table 1.

**Table 1:** The clinicopathological characteristics of GBM patients in TCGA cohort and the relationship between clinicopathological characteristics and S100A9 expression.

**Variables**	**Total (*n* = l60)**	**S100A9**		***X* ^2^**	***p*-value**
		**Low**	**High**		
**OS Status**					
Alive	31 (19.4%)	12 (38.7%)	19 (61.3%)	1.615	0.489
Dead	129 (80.6%)	35 (27.1%)	94 (72.9%)		
**Sex**					
Female	56 (35.0%)	15 (26.8%)	41 (73.2%)	0.278	0.598
Male	104 (65.0%)	32 (30.8%)	72 (69.2%)		
**Age**					
>55	99 (61.9%)	26 (26.3%)	73 (73.9%)	1.152	0.345
≤55	52 (32.5%)	18 (34.6%)	34 (65.4%)		
NA	9 (5.6%)	3 (33.3%)	6 (66.7%)		
**MGMT status**					
Methylated	56 (35.0%)	20 (35.8%)	36 (64.2%)	0.262	0.609
Unmethylated	67 (41.9%)	21 (31.3%)	46 (68.7%)		
NA	37 (23.1%)	6 (16.2%)	31 (83.8%)		
**IDHI status**					
Mutant	9 (5.6%)	7 (77.8%)	2 (22.2%)	10.652	0.001
Wildtype	143 (89.4%)	38 (26.6%)	105 (73.4%)		
NA	8 (5.0%)	2 (25.0%)	6 (75.0%)		
**G-CIMP status**					
G-CIMP	12 (7.5%)	9 (75.0%)	3 (25.0%)	12.871	<0.001
NonG-CIMP	147 (91.9%)	38 (25.9%)	109 (74.1%)		
NA	1 (0. 6%)	0 (0.0%)	1 (100%)		
**Radiotherapy**					
No	21 (13. 1%)	3 (14.3%)	18 (85.7%)	2.744	0.098
Yes	131 (81.9%)	42 (32. 1%)	89 (67.9%)		
NA	8 (5.0%)	2 (25.0%)	6 (75.0%)		
**Chemotherapy**					
No	30 (18.8%)	7 (23.3%)	23 (76.7%)	0.819	0.365
Yes	113 (70.6%)	36 (3 1.9%)	77 (68.1%)		
NA	17 (10.6%)	4 (23.5%)	13 (76.5%)		
**Subtype**					
Classical	39 (24.4%)	15 (38.5%)	24 (61.5%)	0.013	0.005
Mesenchymal	53 (33.1%)	4 (7.5%)	49 (92.5%)		
Neural	28 (17.5%)	6 (21.4%)	22 (78.6%)		
Proneural	38 (23.8%)	21 (55.3%)	17 (44.7%)		
NA	2 (1.3%)	1 (50.0%)	1 (50.0%)		

### Somatic mutation landscape between high- and low-expression groups of S100A9

**Figure 3:**
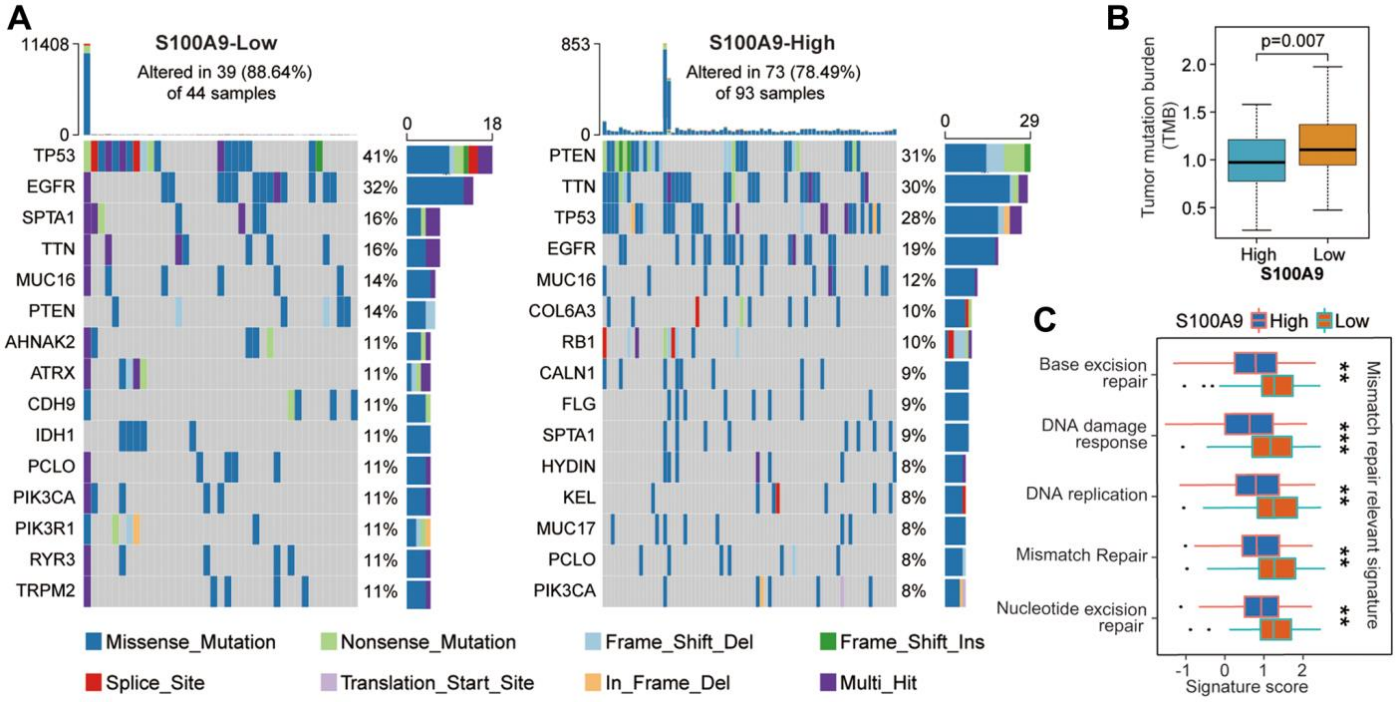
**The difference of tumor somatic mutation landscape between high- and low-expression groups of S100A9.** (**A**) The distribution of the top 15 variants of mutated genes in the high- and low-expression groups of S100A9. The genetic alteration types were listed in the waterfall plots. The upper bar plots represent TMB. The numbers on the right of the bar plots indicated the mutation frequency of each gene. (**B**) Comparison of TMB between high- and low-expression groups of S100A9. (**C**) Differences in mismatch repair-relevant signatures between high- and low-expression groups of S100A9.

Next, we investigated the difference in somatic mutation landscape between S100A9 high- and low-expression groups. By processing and analyzing the mutation annotation files in the TCGA-glioma cohort, the top 15 genes with the highest mutation frequency in the S100A9 high- and low-expression groups displayed in the waterfall plots showed that the mutation frequency and patterns of these genes were significantly different (Figure 3A). Tumor mutational burden (TMB) can reflect the degree of genomic variation of tumor cells, thus indirectly reflecting the ability and degree of the tumor to produce new antigens, and is related to the benefits of immunotherapy [21–23]. Our analysis results showed that compared with the high expression group of S100A9, the patients in the low expression group of S100A9 had higher TMB (Figure 3B) and the scores of mismatch repair relevant signatures (Figure 3C), which to some extent supported the above analysis result that GBM patients in the low expression group of S100A9 had a better prognosis.

### In-depth bioinformatics analysis of S100A9 in GBM

**Figure 4:**
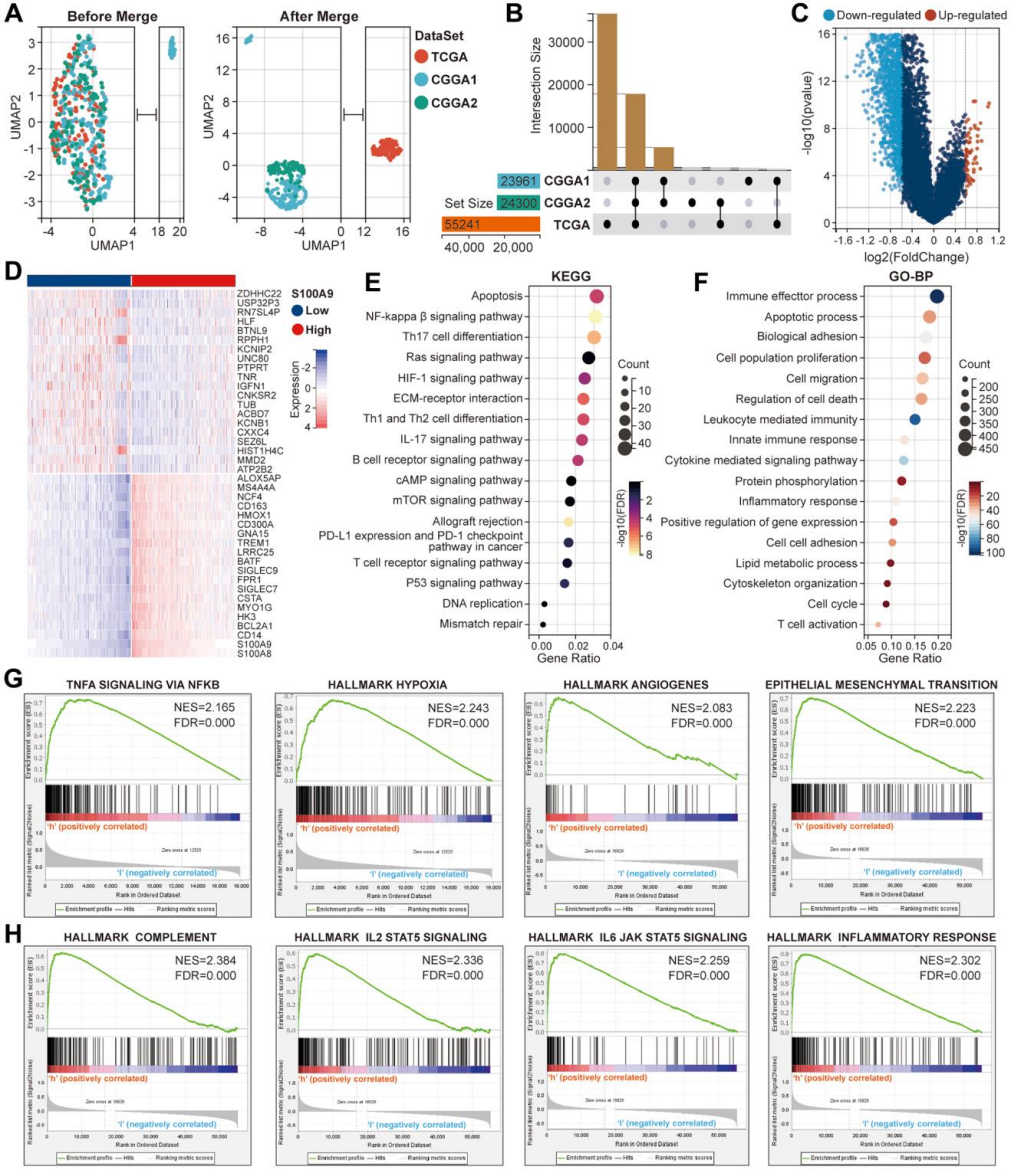
**Further bioinformatics analysis of S100A9 in glioma.** (**A**) Comparison of the distribution of samples in TCGA, CGGA1, and CGGA2 cohorts before and after expression profile merging. (**B**) Presentation of the cross genes in TCGA, CGGA1, and CGGA2 cohorts. (**C**) The volcano plot displayed the DEGs between high- and low-expression groups of S100A9. (**D**) The heat map showed the relative expression levels of the top 40 genes with the most significant difference according to the median expression value of S100A9. (**E**, **F**) The enrichment analysis of KEGG pathways (**E**) and the terms of GO-BP (**F**) based on the DEGs. (**G**, **H**) The gene sets involved in tumor malignant progression (**G**), immune and inflammatory response (**H**) were enriched through differential expression levels of S100A9.

Based on the above studies, to further explore the clinical value of S100A9 in GBM, we made a series of bioinformatics analyses. First, the samples from TCGA (*n* = 160), CGGA1 (*n* = 237), and CGGA2 (*n* = 137) cohorts were merged to obtain a GBM-meta cohort (*n* = 534) by removing the batch effect between different cohorts through the COMBAT algorithm (Figure 4A). Before merging, the samples were scattered, and after merging, the samples were clustered. The number of genes in TCGA, CGGA1, and CGGA2 cohorts before the merger was 55,241, 24,300, and 23,961 respectively, and the number of genes in the GBM-meta cohort after the merger was 173 (Figure 4B). Subsequently, we redivided all the samples into high- and low-expression groups according to the median expression value of S100A9. 5,484 DEGs were identified and displayed in the volcano plot with the screening criteria FDR <0.05 and | log2 (fold change) | >1.3, of which 4,674 were downregulated and 810 were upregulated (Figure 4C). The top 20 genes with the most obvious differences were displayed in the heatmap (Figure 4D). Bubble plots paraded the classical KEGG pathways, enriched by the DEGs, involved in tumor malignant progress, tumor immunity, tumor inflammatory response and gene mutation, such as NF-kappaβ signaling pathway, PD-L1 expression, and PD-1 checkpoint pathway, IL-17 signaling pathway, DNA replication related-pathway, etc., (Figure 4E). Classical GO-BP terms were also paraded in the bubble plots, such as cell migration, apoptotic process, leukocyte-mediated immunity, inflammatory response, etc., (Figure 4F). The well-known gene sets closely related to S100A9, which are involved in tumor malignant progression (Figure 4G) and immune inflammation (Figure 4H), have been screened and shown in the line graphs, such as HALLMARK_HYPOXIA, HALLMARK_ANGIOGENES, HALLMARK_COMPLEMETN, HALLMARK_INFLAMMATORY RESPONSE, etc. The above results indicated that S100A9 was involved in multiple biological processes during the occurrence and development of GBM.

### S100A9 promoted the migration and proliferation of GBM cells *in vitro*

**Figure 5:**
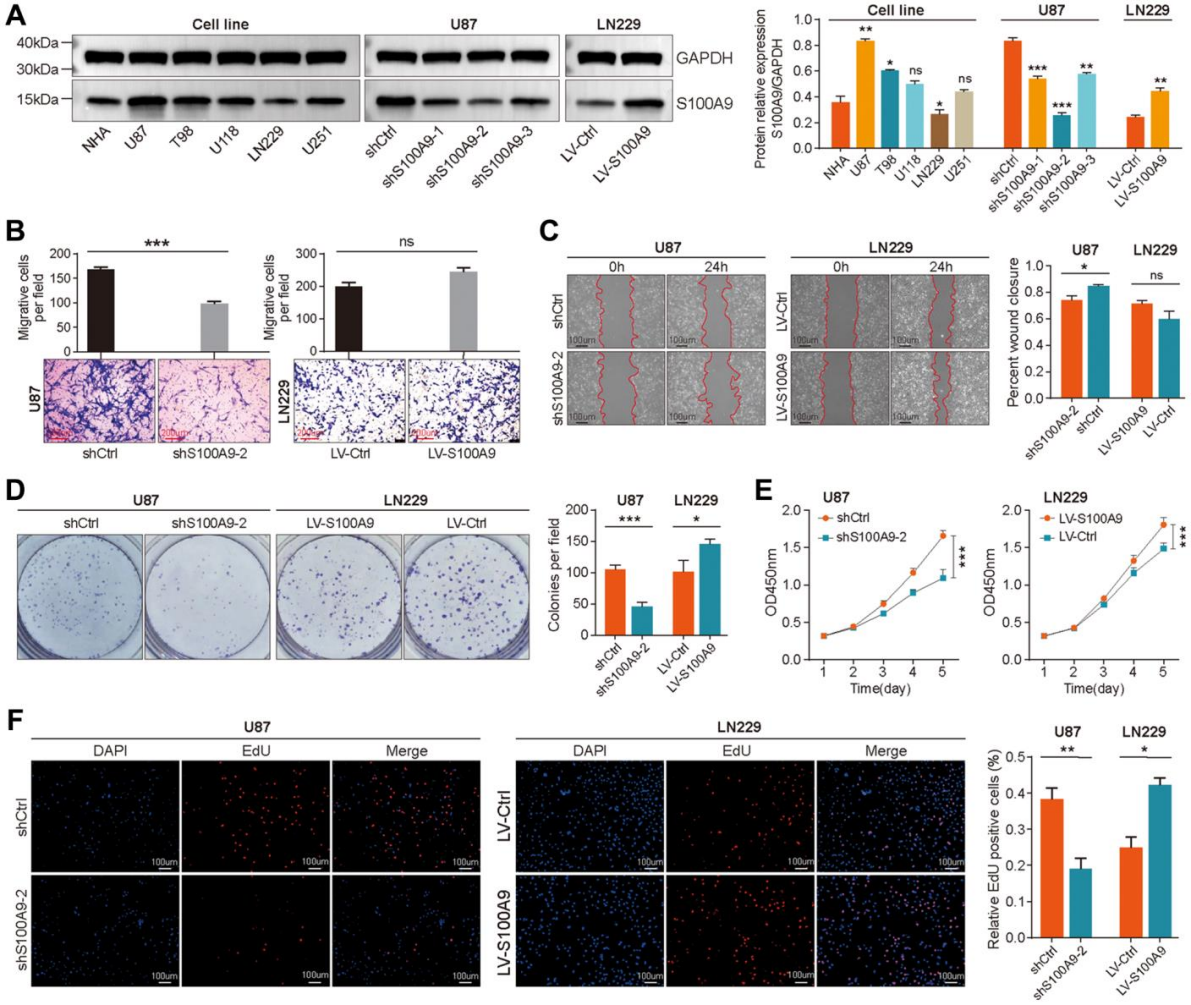
**Upregulation of S100A9 promoted the proliferation of GBM cells.** (**A**) Differential expression of S100A9 protein between human astrocyte cells (NHA) and five GBM cell lines. (**B**, **C**) Transwell assay (**B**) and wound healing assay (**C**) were utilized to detect the migration ability of U87 and LN229 cells transfected with knockout and overexpression S100A9 lentivirus. (**D**–**F**) Colony formation assay (**D**), CCK-8 assay (**E**), and EdU staining (**F**) were utilized to detect the proliferative activity of U87 and LN229 cells transfected with knockout and overexpression S100A9 lentivirus.

Based on bioinformatics analysis, we carried out some cytological experiments *in vitro* to further explore the function of S100A9 in GBM. We selected a human astrocyte cell line (NHA) and five GBM cell lines (U87, T98, U118, LN229, and U251), detected the S100A9 expression level by western blotting and confirmed that S100A9 has the highest expression level in U87 cell line and the lowest expression level in LN229 cell line (Figure 5A). The knockout and overexpression lentiviruses of S100A9 were transfected into U87 and LN229 cell lines respectively, and the transfection effect was detected by western blotting (Figure 5A). We evaluated the migration ability of U87 and LN229 cells post-transfected with lentivirus by transwell assay and wound healing assay and found that S100A9 can promote cell migration to some extent, but its promotion effect is limited (Figure 5B, 5C). The results of the colony formation assay, CCK-8 assay, and EdU staining indicated that the upregulation of S100A9 contributed to the proliferation of U87 and LN229 cells (Figure 5D–5F).

### Immune-related analysis of S100A9 in TCGA-GBM cohort

**Figure 6:**
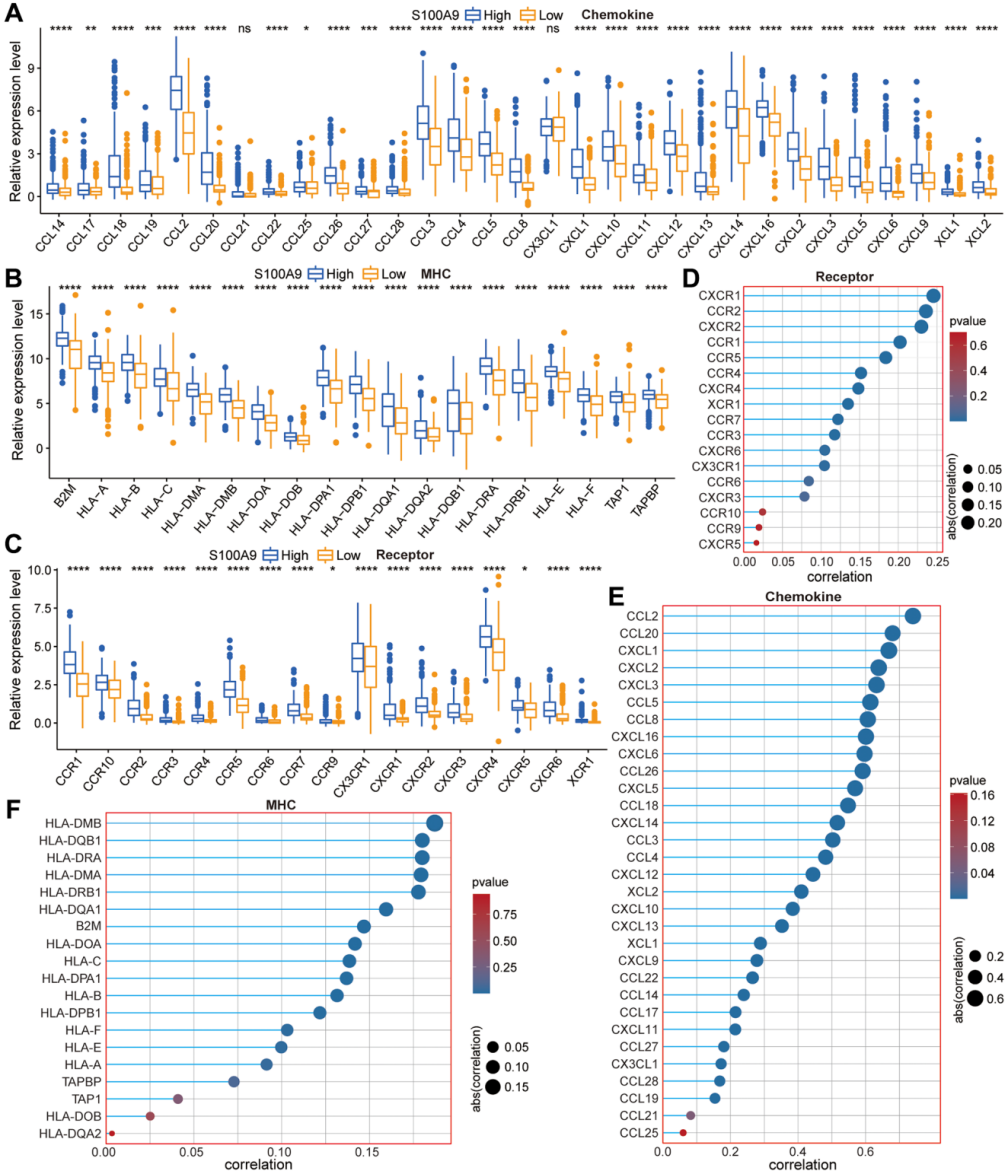
**The relationship between S100A9 and the marker genes of the chemokine pathway, receptor pathway, and MHC pathway.** (**A**–**C**) Differential expression of the marker genes between high- and low-expression groups of S100A9. (**D**–**F**) The correlation between S100A9 and the marker genes.

**Figure 7:**
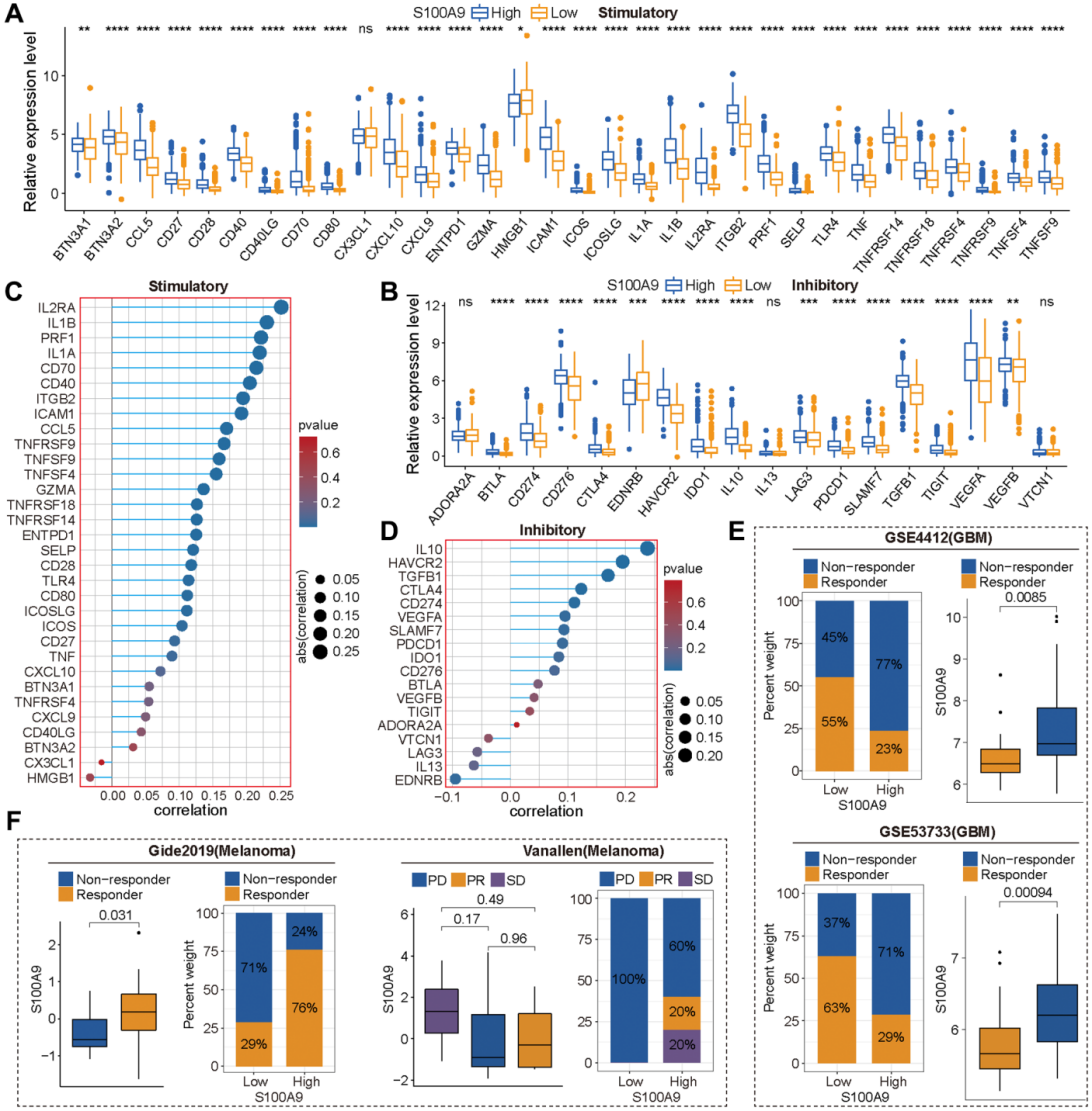
**S100A9 was an effective tool to predict the response of tumor patients to immune checkpoint inhibitors (ICIs).** (**A**, **B**) Differential expression of immune stimulatory genes (**A**) and immune inhibitory genes (**B**) between high and low expression groups of S100A9. (**C**, **D**) The correlation between S100A9 and immune stimulatory genes (**C**) and immune inhibitory genes (**D**). (**E**) In the two glioma cohorts (GSE4412 and GSE53733), S100A9 can effectively predict patients’ responses to ICIs based on the TIED algorithm. (**F**) In the two independent melanoma immunotherapy cohorts (Gide2019 and Vanallen), S100A9 can effectively predict patients’ responses to ICIs. Abbreviations: PD: progressive disease; PR: partial response; SD: stable disease.

The predictive value of immune score and matrix score for tumor microenvironment has been confirmed in multiple tumor types. In GBM, we found that in the high expression group of S100A9, the immune score (Supplementary Figure 1A) and stromal score (Supplementary Figure 1B) were significantly higher than those in the low expression group, which prompted us to conduct further analysis and research. First, we explored the association between marker genes of the chemokine related-pathway, receptor related-pathway, and MHC related-pathway and S100A9, and found that almost all marker genes were differentially expressed between high- and low-expression groups of S100A9 (Figure 6A–6C), and had a significant positive correlation with S100A9 (Figure 6D–6F). This finding prompted us to further explore whether S100A9 could be used as a tool to predict the treatment response of tumor patients to ICIs. The results of differential expression analysis (Figure 7A, 7B) and correlation analysis (Figure 7C, 7D) showed that S100A9 was closely related to immunostimulatory genes and immunoinhibitory genes, which suggested that S100A9 might play a role in the treatment of GBM with ICIs. In two independent GBM cohorts, the TIDE algorithm was used to calculate the TIDE score for each GBM patient, and then they were divided into responder and non-responder groups according to the TIDE score. Further evaluation results indicated that S100A9 could effectively predict the response of GBM patients to ICIs (Figure 7E), which was also verified in two independent melanoma immunotherapy cohorts (Figure 7F).

**Figure 8:**
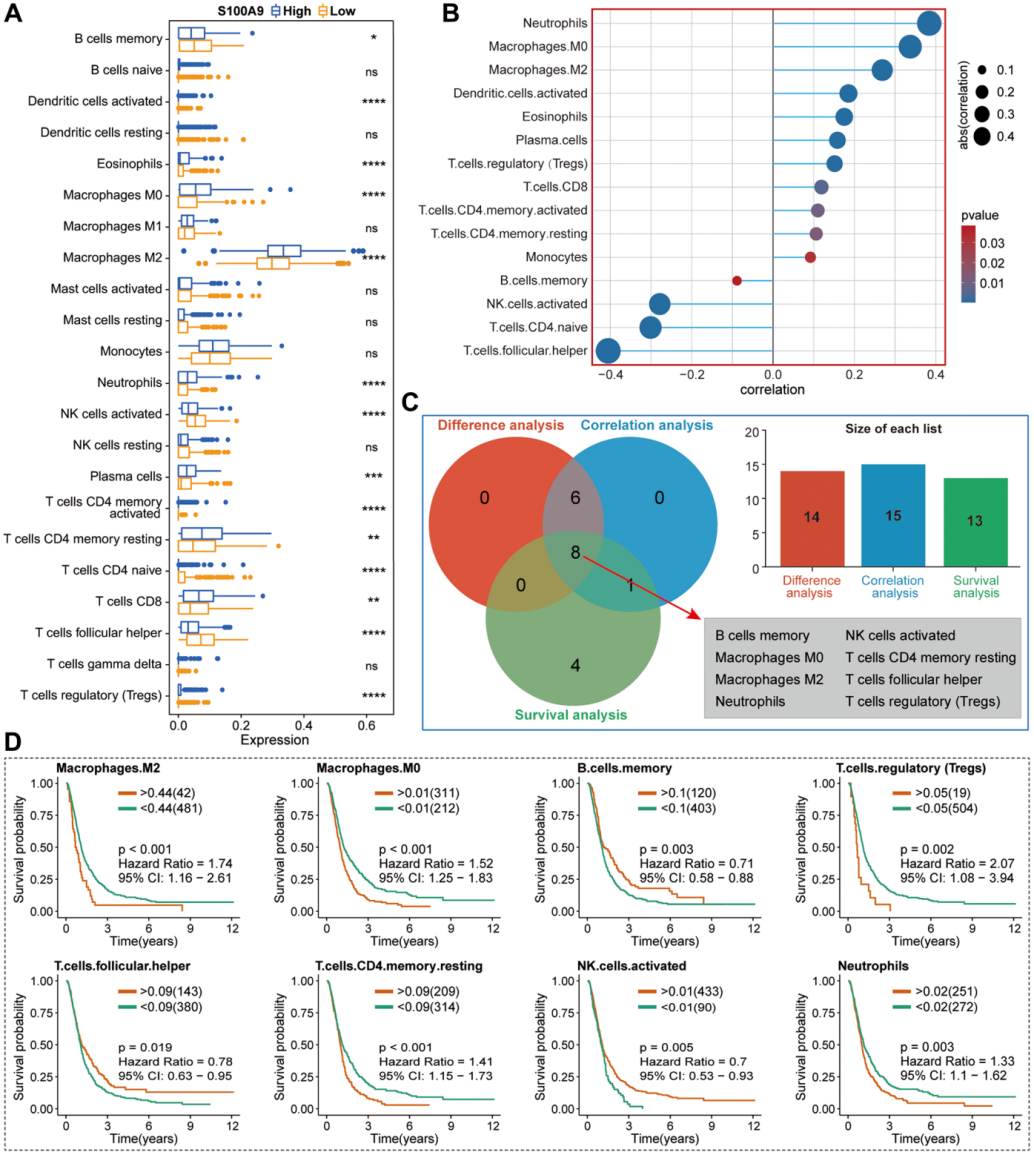
**Basic analysis of TIICs in the TME of GBM in TCGA cohort.** (**A**) Differential analysis of TIICs between high- and low-expression groups of S100A9. (**B**) The correlation between S100A9 and TIICs and the screening criteria was | CC | >0.2 and *p* < 0.05. (**C**) Venn diagram illustrated eight types of TIICs with statistical significance in differential expression analysis, correlation analysis, and survival analysis. (**D**) Survival curves of eight types of TIICs.

**Figure 9:**
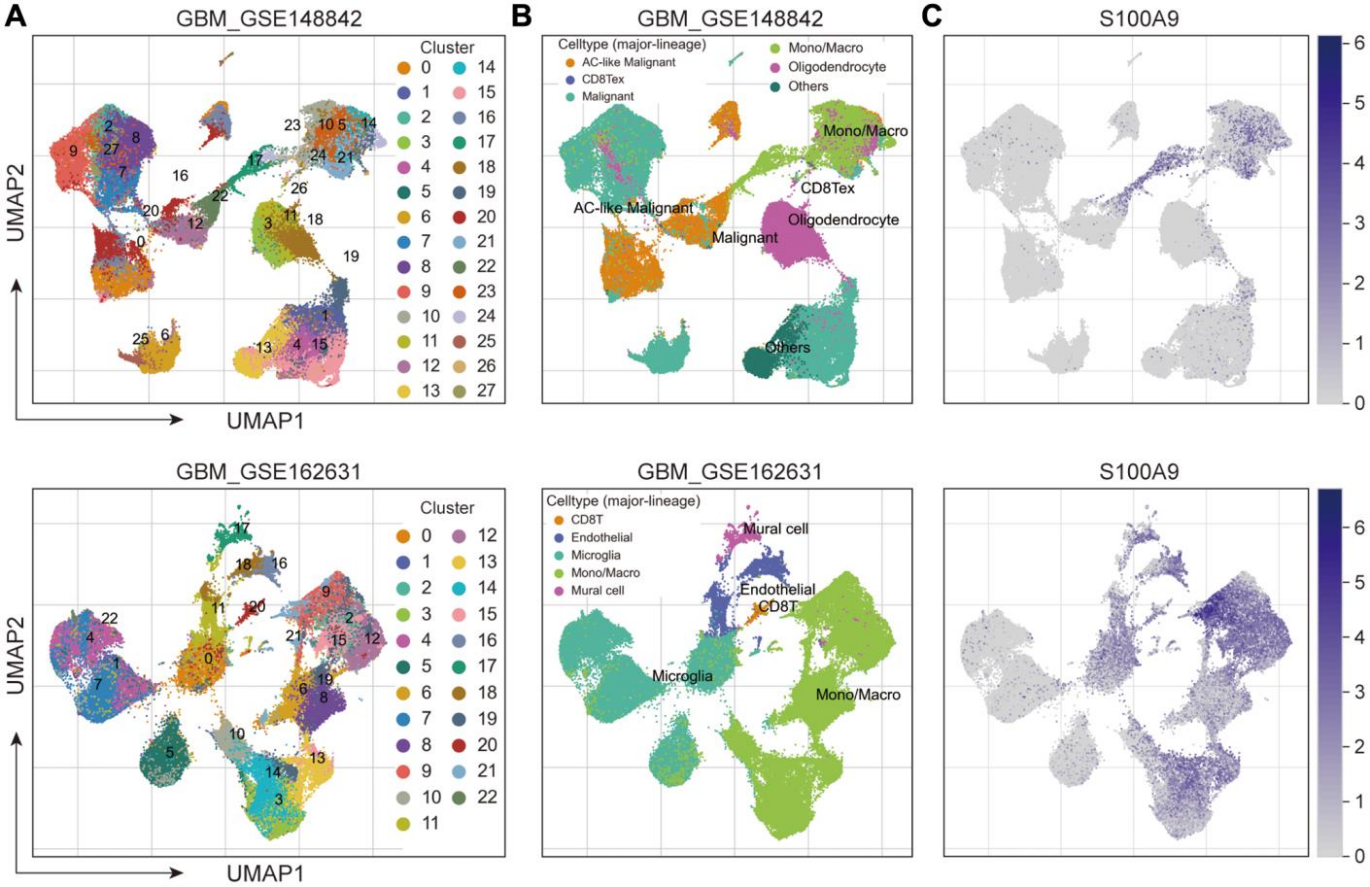
**Exploring the expression and distribution of S100A9 in two sets of GBM scRNA-seq data.** (**A**) Cell clusters were identified in GBM cells and displayed in Uniform Manifold Approximation and Projection (UMAP). (**B**) UMAP plots of different cell types in GBM. (**C**) Expression and distribution of S100A9 in different types of cells.

Then, we explored the TIICs in the TME from three perspectives: the difference analysis of the infiltration levels of TIICs between high- and low-expression groups of S100A9 (Figure 8A), the correlation between the infiltration levels of TIICs and S100A9 (Figure 8B), and the clinical predictive value of TIICs (Figure 8C). Finally, we screened out eight types of TIICs with statistical significance in these three perspectives, namely B cells memory, macrophages M0, macrophages M2, neutrophils, NK cells activated, T cells CD4 memory resting, T cells follicular helper, and T cells regulatory (Tregs) (Figure 8D). Moreover, two sets of scRNA-seq data (GSE_148842 and GSE_162631) were utilized to explore the expression distribution of S100A9 among different types of cells in the TME of GBM (Figure 9A–9C). It was found that S100A9 was mainly expressed in monocytes and macrophages, and only a small amount was expressed in tumor malignant cells.

### S100A9 mediates the migration of M2 macrophages

**Figure 10:**
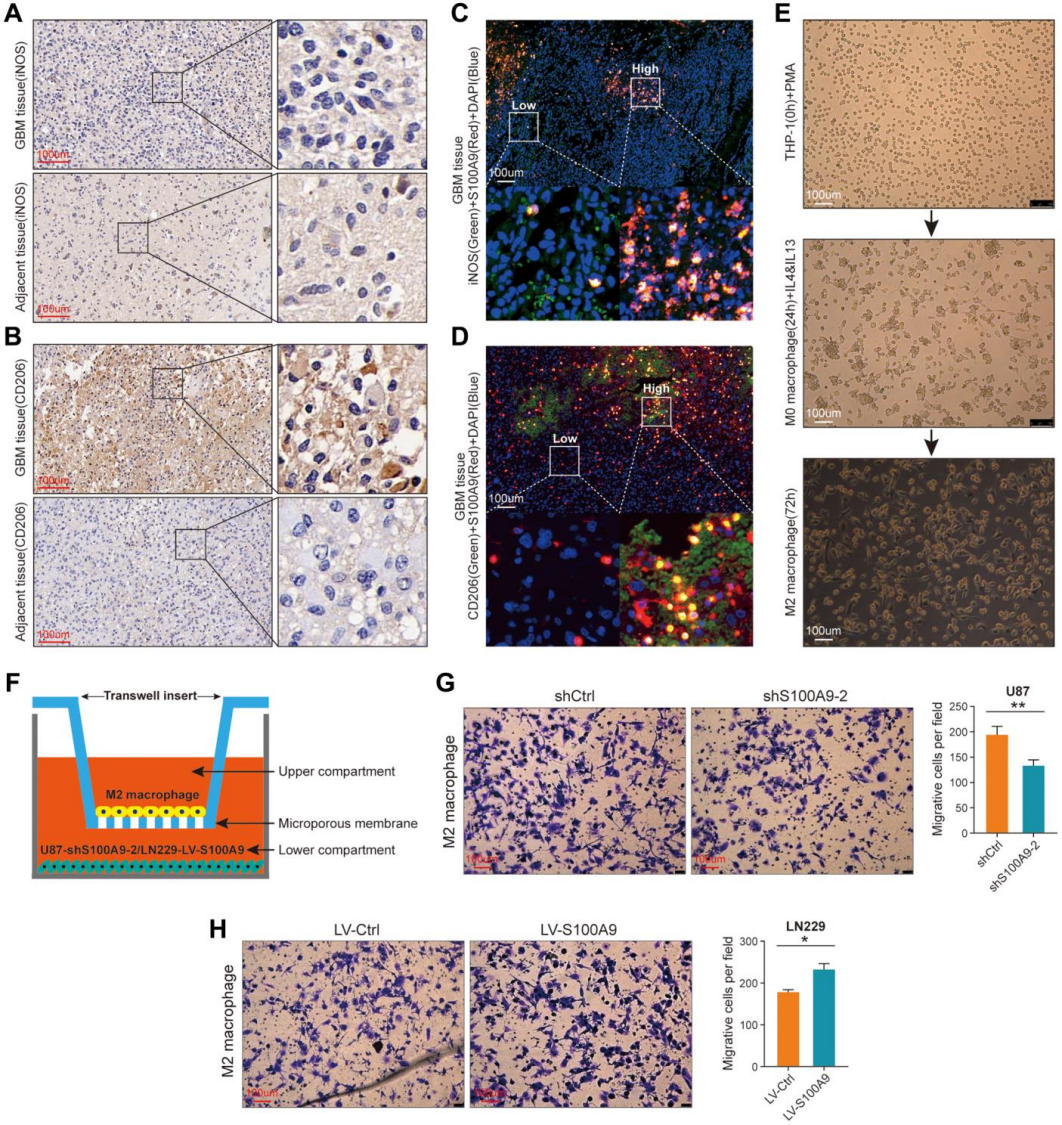
**S100A9 is up-regulated in GBM tissues and contributes to the migration of M2 macrophages.** (**A**, **B**) Immunohistochemical staining of iNOS (**A**) and CD206 (**B**) in GBM and its adjacent tissues. (**C**) Representative image of multiplex immunofluorescence staining of DAPI, iNOS, and S100A9 in GBM tissue. (**D**) Representative image of multiplex immunofluorescence staining of DAPI, CD206, and S100A9 in GBM tissue. (**E**) The process of inducing THP-1 cells into M2 macrophages. (**F**) Sketch map of co-culture of M2 macrophages and U87 cells transfected with knockout S100A9 lentivirus. (**G**, **H**) Transwell assay was utilized to detect the migration ability of M2 macrophages after co-culture with U87 cells transfected with lentivirus knockout S100A9 and LN229 cells transfected with lentivirus overexpressing S100A9.

In IHC staining, the expression level of iNOS protein reflecting the infiltration level of M1 macrophages in GBM tissues is not significantly increased compared with its corresponding adjacent tissues (Figure 10A), while the expression level of CD206 protein reflecting the infiltration level of M2 macrophages in GBM tissues is significantly increased compared with its corresponding adjacent tissues (Figure 10B). In Figure 8A, 8B, the infiltration level of M2 macrophages was significantly higher than that of other types of TIICs, and there was a positive correlation between M2 macrophages and SA100A9, which is not found in M1 macrophages. Immunofluorescence staining showed that S100A9 protein had no obvious correlation with iNOS protein (Figure 10C), but had a certain positive correlation with CD206 protein (Figure 10D), which was consistent with the above bioinformatics analysis results. Finally, we further explored whether S100A9 could promote the migration of M2 macrophages through cytological experiments *in vitro*. Human THP-1 cells were induced to differentiate into M2 macrophages by adding biological inducers (Figure 10E), and then M2 macrophages were co-cultured with U87 cells transfected with knockout shS100A9-2 lentivirus using a transwell device (Figure 10F) to investigate whether S100A9 could promote the migration of M2 macrophages. The results showed that U87 cells with down-regulated S100A9 inhibited the migration of M2 macrophages (Figure 10G). On the contrary, LN229 cells with up-regulated S100A9 can promote the migration of M2 macrophages (Figure 10H). In a word, these findings indicated that S100A9 was involved in mediating the migration of M2 macrophages.

## DISCUSSION

In this study, we carried out a large-scale bioinformatics analysis and *in vitro* cytological experiments on S100A9 in GBM and finally got several main findings:

(1) S100A9 is up-regulated in GBM tissues and is associated with poor prognosis, and can be used as an independent prognostic factor in GBM patients; (2) S100A9 can be used as an effective tool to predict the immunotherapy response for GBM patients; (3) S100A9 can promote the malignant progression of GBM; (4) S100A9 is mainly expressed in monocytes and macrophages, and the modest amount is also expressed in malignant tumor cells; (5) S100A9 contributes to the migration of M2 macrophages.

Our findings indicate that S100A9 is a potential biomarker and therapeutic target for GBM.

As the main cell populations of TME, immune cells are actively involved in various stages of cancer development, for instance, tumor occurrence, progression, recurrence, etc., [24, 25]. The expression of S100A9 is found in multiple cell types, which triggers various signaling pathways related to cell biological processes by binding with cell surface receptors, such as cell cycle, cell survival, cell differentiation, etc., [14]. Numerous evidences indicated that S100A9 is up-regulated in many types of tumors [26]. In addition, due to the fact that S100A9 protein not only exists in cancer cells but can also be secreted into the extracellular environment, its potential as a promising biomarker for tumor diagnosis or prognosis prediction has received widespread attention. Our study found that S100A9 was also significantly up-regulated in GBM tissues, mainly expressed in monocytes and macrophages, and also expressed in malignant tumor cells. Moreover, the expression of S100A9 mRNA has prognostic significance for GBM patients.

S100A9 produces stable homodimers or heterodimers with other members of the S100 family by changing its conformation, which is related to multiple signaling pathways. These cascade pathways control immune homeostasis and cellular metabolism, which often transition into specific states to promote tumor growth [27, 28]. To further study the function of S100A9 in GBM, we conducted GO and KEGG enrichment analysis on the DEGs screened from high- and low-expression groups of S100A9 and found that S100A9 participated in a variety of pathways and biological processes related to tumor progression and immune regulation. The enrichment results of GSEA also highlighted some obvious HALLMARK gene sets related to tumor progression and immune regulation. To verify the results of bioinformatics analysis, we carried out some cytological experiments *in vitro*. U87 and LN229 cell lines were used to construct stable knockout and overexpression S100A9 cell models by transfecting lentivirus. The results confirmed that the increased expression of S100A9 in GBM cells enhanced the malignant phenotype of GBM cells, especially the proliferation of GBM cells.

Without the influence of immune cells, S100A9, as a calprotectin, may induce changes in cell function through its impacts on the internal environment of cells. Meanwhile, to verify the exocrine effect of S100A9 in GBM cells, we conducted an immune analysis in the TCGA cohort and found the expression of S100A9 was positively correlated with most of the immune markers. Moreover, S100A9 was found in two GBM cohorts and two melanoma cohorts as a robust tool for predicting cancer patients’ response to ICIs. In the TME of GBM, we found that M2 macrophages had the highest infiltration level and were positively correlated with S100A9 expression. In addition, the analysis of two sets of gliomas scRNA-seq data illustrated that S100A9 was mainly expressed in monocytes and macrophages. These findings prompted us to further investigate the relationship between S100A9 and M2 macrophages. From the perspective of GBM tissue sections, we found that M2 macrophages changed most significantly with the expression of S100A9 in tissues, also showing a positive correlation distribution trend, and suggesting that S100A9 may have a recruitment effect on M2 macrophages or promoting the polarization of macrophages to M2. To further verify the hypothesis, we conducted a cell co-culture experiment. The results showed that M2 macrophages were more inclined to the environment with a higher level of S100A9. Previous research on S100A9 in tumors focused on immune-related cells and paracrine function while ignoring the effect of S100A9 on tumor cells themselves. The above experimental results proved that S100A9 had both the ability to affect the malignant phenotype of GBM cells and change the extracellular microenvironment. This conclusion further enhanced the importance of S100A9 as a potential therapeutic target in the treatment of GBM.

Previous studies have suggested that a slightly higher extracellular S100A9 level can promote the development of tumors, but a higher S100A9 level can induce apoptosis of tumor cells; The concentration of S100A9 in cells may affect the epithelial-mesenchymal transformation signal [29]. In our study, we did not find cell apoptosis, which may be due to the limited amount of exocrine secretion after overexpression of S100A9 by tumor cells or the intensity of GBM cells to induce apoptosis of S100A9. In addition, the biological process of epithelial-mesenchymal transformation in GBM is quite different from that in other tumors [30]. Therefore, the research results of S100A9 in other types of tumors may not be fully applied to GBM cells and the specific effect of S100A9 in GBM needs further exploration.

This study also has some limitations. We are unable to build a complete immune microenvironment model, and instead of reaching from the perspective of tumor cells and M2 macrophages. Although the variables were simple and the results were effective, we cannot exclude the unpredictable effects caused by the complex tumor microenvironment. Moreover, it is difficult to completely distinguish the effect of S100A9 in the intracellular and extracellular, which needs further experimental verification. In future work, we will conduct in-depth exploration from multiple perspectives such as biological mechanisms, drug targets, and biological model construction, in order to provide effective assistance for GBM treatment.

## CONCLUSIONS

On the whole, this study not only highlights the potential value of S100A9 in predicting prognosis and immunotherapeutic response in GBM patients but also clarifies that S100A9 can promote the malignant progression of GBM and enhance the migration of M2 macrophages.

## MATERIALS AND METHODS

### Data collection and preprocessing

Twenty-five pairs of GBM tissues (World Health Organization grade IV) and adjacent non-tumor tissues were collected from Zhoukou Central Hospital. The expression data and corresponding clinical data of GBM samples were downloaded from the TCGA database (*N* = 160) (https://portal.gdc.cancer.gov/) and CGGA database (CGGAseq1, *N* = 237; CGGAseq2, *N* = 137) (http://www.cgga.org.cn/). Other GBM cohorts included in this study were obtained from the Data Visualization Tools for Brain Tumor Datasets (GlioVis, http://gliovis.bioinfo.cnio.es/). The unified standardized pan-cancer dataset (*N* = 19131, G = 60499) was downloaded from the UCSC database (https://xena.ucsc.edu/) and the expression data was transformed with log2 (x + 0.001). If the sample size of a cancer species is less than three, it will not be enrolled in further analysis.

### Basic analysis of S100A9

Differential expression analysis and prognosis analysis of S100A9 from the perspective of pan-cancer was completed on the Biomedical data analysis box (SangerBox3.0, http://vip.sangerbox.com/home.html). The GEPIA2 website (http://gepia.cancer-pku.cn/) was used to conduct the differential expression analysis of S100A9 in the TCGA cohort. The R packages “ggpubr” (version 0.4.0) and “ggplot2” (version 3.3.3) were applied to analyze the differential expression of S100A9 in four GBM cohorts. Kaplan-Meier (K-M) survival curves were plotted for S100A9 in six GBM cohorts by using the R packages “surviver” (version 0.4.9) and “survival” (version 3.3-1). Univariate and multivariate Cox regression analysis was performed to explore the independent prognostic value of S100A9 in GBM patients by using the R packages “surviver” and “survival”.

### Investigation of tumor somatic mutation landscape

The R package “TCGAbiolinks” was employed to download the somatic mutation data from the Genomic Data Commons (GDC) [31]. MuTect2 algorithm [32] was employed to process somatic mutation data. The top 15 genes with the highest mutation frequency were extracted and presented in the form of a waterfall using the R package “maftools” (version 2.6.05). Using Strawberry Perl (version 5.30.1) to calculate the tumor mutation burden (TMB) for GBM patients based on the somatic mutation data. The calculation of mismatch repair relevant signature scores for GBM patients refers to the study of Zeng et al. [33].

### Signaling pathway and functional enrichment analysis

Due to the limited number of GBM samples contained in a single cohort, we merged TCGA, CGGA1, and CGGA2 cohorts into a meta-glioma cohort using the COMBAT algorithm on the SangerBox3.0 platform [34]. Differentially expressed genes (DEGs) were screened with | log2 (fold change) | >1.3 and FDR <0.05 based on the median expression value of S100A9 in the meta-GBM cohort by using the R packages “DESeq2” (Version 1.26.0) and “limma” (version 3.46.0) [35, 36]. The enrichment of the KEGG signaling pathways and the terms of GO-BP was carried out by using the R package “clusterProfiler” (Version 3.18.1) 22455463. Gene set enrichment analysis (GSEA) was conducted by using the GSEA software (4.2.3).

### Immune-related analysis

Immune stimulatory and inhibitory genes, marker genes of the chemokine pathway, receptor pathway, and MHC pathway, were searched from previous studies [37, 38]. CIBERSORT algorithm was applied to calculate the enrichment scores of different types of tumor immune infiltrating cells (TIICs) in the TME, which revealed the infiltration levels of TIICs [39]. Based on the gene expression profiles of the tumor before treatment and the mechanisms of induction of T cell dysfunction and prevention of T cell infiltration in tumors, the Tumor Immune Dysfunction, and Exclusion module (TIDE, http://tide.dfci.harvard.edu/) can predict tumor patients’ response to ICIs [40]. Tumor samples highly correlated with T cell infiltration were classified as responders, otherwise, they were classified as non-responders. The R packages “limma” and “reshape2” (version 1.4.4) are used to perform differential expression analysis of multiple continuous variables between two groups. The correlation between continuous variables was explored by the R package “corrplot” (version 0.92). The transcriptome data and clinical data of two melanoma cohorts (Gide2019 and Vanallen) that have received immunotherapy are downloaded from TIDE website [41] to evaluate the application value of S100A9 in predicting clinical immunotherapy response.

### Single-cell RNA sequencing (scRNA-seq) analysis

Two sets of scRNA-seq data, GBM-GSE148842 and GBM-GSE162631, were downloaded from the Tumor Immune Single-cell Hub 2 (TISCH2) (http://tisch.comp-genomics.org/). Uniform Manifold Approximation and Projection (UMAP) technique was used to determine cell clusters [42]. The document column of the TISCH2 website introduced the data processing process [43].

### Cell culture and transfection

The Chinese Academia Sinica Cell Repository provided us with cell lines. Except human monocyte THP-1 was cultured in RPMI-1640 medium (Gibco, China), other cell lines were cultured in MEM (Gibco, China) or DMEM (Gibco, China) medium, and the medium contained 10% FBS (ExCell, China), 100 μg/ml streptomycin (Gibco, USA) and 100 U/ml penicillin (Gibco, USA), and the cell incubator was maintained at 37°C and 5% CO_2_. The knockout and overexpression lentivirus of S100A9 were constructed by the Sheweisi Biotechnology Company (Tianjin, China) using U6-MCS-CMV-zsGreen-PGK-Puromycin and CMV-MCS-3FLAG-SV40-mCherry-IRES-Puromycin respectively. The sequence of S100A9 shRNA was as follows: shS100A9-1, 5′-CATCAACACCTTCCACCAATA-3′, shS100A9-2, 5′-ATGGAGGACCTGGACACAAAT-3′, and shS100A9-3, 5′-TCAAGAAGGAGAATAAGAATG-3′. For lentivirus transfection, refer to the instructions provided by the Sheweisi Biotechnology Company.

### Protein extraction and western blotting

The S100A9 rabbit polyclonal antibody (26992-1-AP, Proteintech, China), GAPDH rabbit polyclonal antibody (60004-1-Ig, Proteintech), and Beta Tubulin rabbit polyclonal antibody (10068-1-AP, Proteintech) were used as primary antibodies. Goat anti-rabbit IgG (SA00001-2, Proteintech) was used as the second antibody. The dilution ratio of antibodies was executed according to the corresponding instructions. Next, we will describe the extraction process of tissue protein. First, the tissue was weighed and cut, and put into a 2 ml EP tube. High-efficiency radioimmunoprecipitation assay (RIPA) buffer and phenylmethanesulfonyl fluoride (PMSF, R0020, Solarbio, China) were mixed in the proportion of 100:1 to prepare tissue lysate. 1 ml lysate was added to every 50 mg of tissue and homogenized, and then split on ice for 30 minutes. Finally, the tissue was centrifuged for 10 minutes with a high-speed centrifuge at 4°C and 12000 rpm. After discarding the tissue precipitation, 5× loading buffer solution was added to the upper clear solution, and then boil it about five minutes for later experiments. As for the extraction of cell protein, the first step is to collect cells and wash them with PBS twice. The subsequent steps are the same as the extraction of tissue protein. The operation process of western blotting refers to our previous study [44].

### Immunohistochemical and immunofluorescence staining

The tissues were fixed with 10% formalin for one week, then embedded with paraffin and sectioned (four-micrometer). The tissue sections were deparaffinized and dehydrated and treated with 3% hydrogen peroxide for about 10 minutes. After that, the primary antibody against S100A9 was used to stain tissues at 4°C for one night after blocking with 5% BSA for about one hour at RT. Then, the tissue sections were treated with secondary antibody at RT for one hour, followed by DAB staining, target molecules detection, and hematoxylin counterstaining in turn. For immunofluorescent staining, tissue sections were immunostained with primary antibodies against S100A9, iNOS (22226-1-AP, Proteintech), and CD206 (18704-1-AP, Proteintech) overnight at 4°C, and then incubated with fluorochrome-conjugated antibodies. After that, DAPI was added as a nuclear counterstain. A fluorescent microscope (Leica, Germany) was applied to collect the final image.

### Cell migration assay

First, 150 μl serum-free medium and 700 μl medium containing 15% FBS were added to the upper and lower transwell chambers (Corning, USA) respectively. Then, 8 × 10^4^ cells transfected with lentivirus were seeded in the upper chamber. After 24 hours of incubation, a microcellular scraper was used to remove the cells remaining in the upper chamber. 4% ice-precooled paraformaldehyde (Solarbio, P1110) was applied to fix the bottom of the upper chamber for 30 minutes, washed with phosphate-buffered saline (PBS), stained with 0.1% crystal violet (Solarbio, G1075) for 20 minutes. Finally, the chambers were washed several times, dried, and photographed with a microscope (Leica, Germany). The number of cells was calculated by ImageJ Software (Version 1.8.0.345).

When the cell adhesion concentration in the six-well plates (Corning, USA) was 80–90%, a 200 μl sterile spear was used to cut through the bottom of the plate to create an artificial wound. After washing the floating cells, the adherent cells were continued cultured in a serum-free medium. At 0 and 24 hours, wound closure was photographed using an inverted Leica microscope. The wound closure at 0 and 24 hours was recorded by a camera (KONKA, China). The wound area was calculated by ImageJ Software.

### Cell proliferation assay

Cells were seeded into 6-well plates at 1000 cells/well and cultured for about 15 days. It was predicted that the cells would proliferate for about 5–7 generations. The culture medium was changed every three days, and the colony formation was closely observed. Cell culture was stopped when the number of cells in a single colony approached 50. The colonies were fixed with 4% ice-precooled paraformaldehyde for about 20 minutes, then stained with 0.1% crystal violet for about 20 minutes.

The Cell Counting Kit-8 (CCK-8, Beyotime, Shanghai) was employed to assay cell proliferation activity. Cells were seeded into 96-well plates at 2000 cells/well. Adding 10 ul CCK-8 reagent to each well and continuing to culture in the incubator for two hours, the absorbance of cells was measured at 450 nm every 24 hours for 5 consecutive days.

2 × 10^4^ cells were seeded into each well of the 24-well plate and cultured until the cell adhesion concentration reached about 75%. BeyoClick™ EdU Cell Proliferation Kit (Beyotime, Shanghai) was used to measure cell proliferation activity according to the manufacturer’s instructions. The nuclei of all cells with blue fluorescence and the positive cells with red fluorescence were photographed by fluorescence microscopy, and the results were analyzed by ImageJ Software.

### Induction of human monocyte HTP-1

THP-1 cells treated with Phorbol 12-myristate 13-acetate (PMA, 150 ng/ml, Absin^®^, China) for 6–8 hours can be induced to differentiate into M0 macrophages, which attach to the bottom of the culture dish. After removing the non-adherent cells by discarding the medium and washing with PBS, the medium containing PMA (150 ng/ml), IL-4 (20 ng/ml), and IL-13 (20 ng/ml) was added and continued to culture for about 48 hours, and the M0 macrophages would be induced to differentiate into M2 macrophages.

### Migration activity of M2 macrophages

The co-culture system was conducted in the transwell chambers and 24-well plates (Corning, USA). The shCtrl and shS100A9-2 groups of U87 cells were seeded into the lower chamber at 1 × 10^5^ cells/chamber, respectively, and 1 × 10^5^ M2 macrophages were seeded into the upper chamber. After 30 hours of co-culture, the following operations of cell fixation, staining, photography, and data processing are described above.

### Statistical analyses

R software (version 4.0.3) and GraphPad Prism software (version 8.0.2) were applied for statistical analysis. Survival analysis was assessed by K–M curves with a log-rank test. Independent prognostic factors were identified by univariate and multivariate Cox regression analysis. The unpaired Student’s *t*-test and Mann–Whitney *U*-test were used to evaluate the statistical differences of normally and non-normally distributed continuous variables, respectively. The Fisher’s exact test or chi-square test was carried out to analyze the statistical significance of differences between categorical variables. The Spearman analysis method was performed to estimate correlation coefficients between two continuous variables. All experiments were independently repeated three times. Recognition criteria for statistical differences: ^*^p < 0.05, ^**^p < 0.01, ^***^p < 0.001, and ^****^p < 0.0001.

### Availability of data and materials

Data associated with this study are summarized in the manuscript or included in the supplemental information.

## Supplementary Materials

Supplementary Figure 1

Supplementary Table 1
