# Supplementary material for: S100A9, as a potential predictor of prognosis and immunotherapy response for GBM, promotes the malignant progression of GBM cells and migration of M2 macrophages
Source: Aging (Albany NY). 2024 Aug 13;16(15):11513–34. doi: 10.18632/aging.205949 (PMC11346789; doi:10.18632/aging.205949)
Supplement: Supplementary Figure 1 [file aging-16-205949-s001.pdf]

SUPPLEMENTARY FIGURE

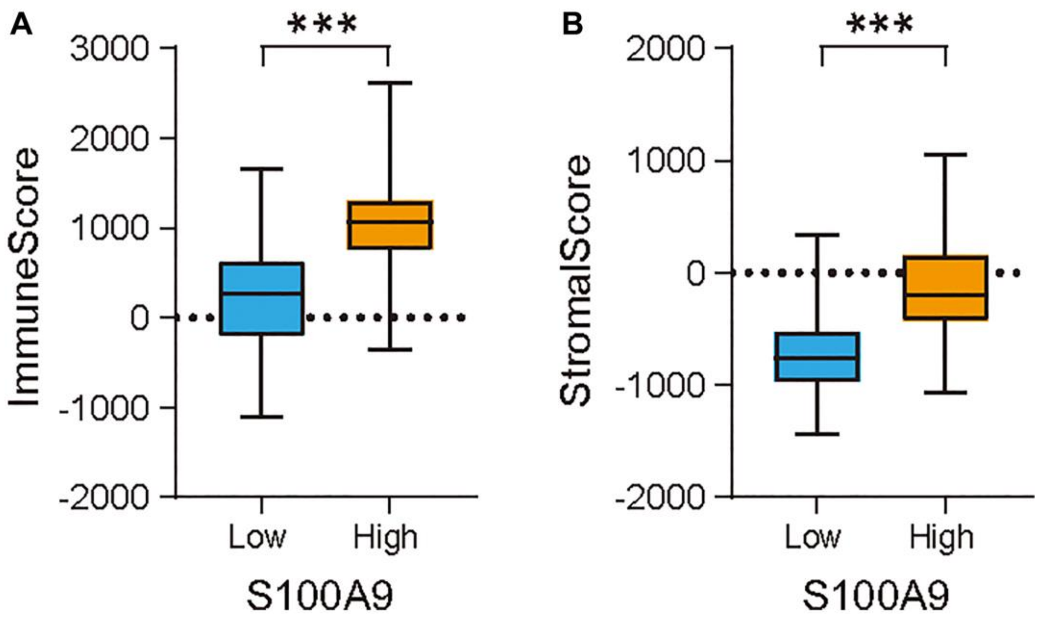

Supplementary Figure 1. Differential analysis of immune score and stromal score between high and low expression groups of S100A9. (**A**) The immune score of the S100A9 high expression group was significantly higher than that of the S100A9 low expression group. (**B**) The stromal score of the S100A9 high expression group was significantly higher than that of the S100A9 low expression group.
