# Supplementary material for: S100A9, as a potential predictor of prognosis and immunotherapy response for GBM, promotes the malignant progression of GBM cells and migration of M2 macrophages
Source: Aging (Albany NY). 2024 Aug 13;16(15):11513–34. doi: 10.18632/aging.205949 (PMC11346789; doi:10.18632/aging.205949)
Supplement: Supplementary Table 1 [file aging-16-205949-s002.pdf]

SUPPLEMENTARY TABLE

Supplementary Table 1. Abbreviations of various cancers in pan-cancer.

**ACC**	Adrenocortical carcinoma
**BLCA**	Bladder Urothelial Carcinoma
**BRCA**	Breast invasive carcinoma
**CESC**	Cervical squamous cell carcinoma and endocervical adenocarcinoma
**CHOL**	Cholangiocarcinoma
**COAD**	Colon adenocarcinoma
**ESCA**	Esophageal carcinoma
**GBM**	Glioblastoma multiforme
**HNSC**	Head and Neck squamous cell carcinoma
**KICH**	Kidney Chromophobe
**KIRC**	Kidney renal clear cell carcinoma
**KIRP**	Kidney renal papillary cell carcinoma
**LAML**	Acute Myeloid Leukemia
**LGG**	Brain Lower Grade Glioma
**LIHC**	Liver hepatocellular carcinoma
**LUAD**	Lung adenocarcinoma
**LUSC**	Lung squamous cell carcinoma
**OV**	Ovarian serous cystadenocarcinoma
**PAAD**	Pancreatic adenocarcinoma
**PRAD**	Prostate adenocarcinoma
**READ**	Rectum adenocarcinoma
**SKCM**	Skin Cutaneous Melanoma
**STAD**	Stomach adenocarcinoma
**TGCT**	Testicular Germ Cell Tumors
**THCA**	Thyroid carcinoma
**UCEC**	Uterine Corpus Endometrial Carcinoma
**UCS**	Uterine Carcinosarcoma
**COADREAD**	Colon adenocarcinoma/Rectum adenocarcinoma Esophageal carcinoma
**ALL**	Acute Lymphoblastic Leukemia
**STES**	Stomach and Esophageal carcinoma
**KIPAN**	Pan-kidney cohort (KICH+KIRC+KIRP)
**WT**	High-Risk Wilms Tumor
**PCPG**	Pheochromocytoma and Paraganglioma
